# An Extended ΔCT-Method Facilitating Normalisation with Multiple Reference Genes Suited for Quantitative RT-PCR Analyses of Human Hepatocyte-Like Cells

**DOI:** 10.1371/journal.pone.0093031

**Published:** 2014-03-21

**Authors:** Gesa Riedel, Urda Rüdrich, Nora Fekete-Drimusz, Michael P. Manns, Florian W. R. Vondran, Michael Bock

**Affiliations:** 1 REBIRTH Research Group “Hepatic Cell Therapy”, Hannover Medical School, Hannover, Germany; 2 Department of Gastroenterology, Hepatology and Endocrinology, Hannover Medical School, Hannover, Germany; 3 Regenerative Medicine & Experimental Surgery (ReMediES), Department of General, Visceral and Transplantation Surgery, Hannover Medical School, Hannover, Germany; Inserm, U1052, UMR 5286, France

## Abstract

Reference genes (RG) as sample internal controls for gene transcript level analyses by quantitative RT-PCR (RT-qPCR) must be stably expressed within the experimental range. A variety of *in vitro* cell culture settings with primary human hepatocytes, and Huh-7 and HepG2 cell lines, were used to determine candidate RG expression stability in RT-qPCR analyses. Employing GeNorm, BestKeeper and Normfinder algorithms, this study identifies *PSMB6, MDH1* and some more RG as sufficiently unregulated, thus expressed at stable levels, in hepatocyte-like cells *in vitro*. Inclusion of multiple RG, quenching occasional regulations of single RG, greatly stabilises gene expression level calculations from RT-qPCR data. To further enhance validity and reproducibility of relative RT-qPCR quantifications, the ΔCT calculation can be extended (e-ΔCT) by replacing the CT of a single RG in ΔCT with an averaged CT-value from multiple RG. The use of two or three RG - here identified suited for human hepatocyte-like cells - for normalisation with the straightforward e-ΔCT calculation, should improve reproducibility and robustness of comparative RT-qPCR-based gene expression analyses.

## Introduction

The quantification of mRNA expression levels by reverse transcription quantitative PCR (RT-qPCR) is - if applied appropriately [1] - a powerful and straightforward tool. Most RT-qPCR analyses employ reference genes (RG), i.e. genes that are stably expressed within the experimental range, for normalisation purposes. Whereas for other tissues appropriate RG have already been verified [2]–[4], to our knowledge no in-depth analysis has been performed for human hepatocyte-like cells *in vitro* so far.

A literature survey covering eight volumes of the journal HEPATOLOGY (years 2009–2012) for RT-qPCR-analyses of human hepatocyte-like cells – i.e. primary hepatocytes and hepatic cell lines - identified *GAPDH* as the most commonly used RG (in 79 of 192 papers, i.e. 41%), followed by *ACTB* (46/192, 24%). Also, of the 192 RT-qPCR-papers found, 91 analysed primary liver material from patients and 131 studies included hepatic cell lines, wherein HepG2 (59%) and Huh-7 (47%) cell lines were by far the most commonly used ones.

Hepatocytes and hepatoma-derived cell lines are used for a plethora of *in vitro* investigations mainly in the areas of basic, preclinical and pharmacological research - including metabolic assays, drug- and drug-toxicity testings, accompanied by optimisations of culture conditions towards enhanced cell maturity. Also, studies with infectious human hepatotropic agents are of major importance and more recently, *in vitro* gene therapeutic approaches using primary hepatocytes - requiring the issues of rapid dedifferentiation and almost complete absence of proliferation of primary hepatocytes in tissue culture to be overcome, possibly by the culture of proliferating hepatic stem cells and their subsequent maturation in the future.

Investigating the expression stability of 22 widely used RG in experimental *in vitro* settings with commonly used hepatocyte-like cell types, we identified in this study several reference genes suited for RT-qPCR-analyses of hepatocyte-like cells. Furthermore we were able to derive from the ΔCT-method a simple calculation allowing for the inclusion of multiple RG to significantly strenghten data normalisation in comparative, not absolute, gene expression analyses - without the need for laborious generation of qPCR-standard curves.

## Materials and Methods

A more detailed description of materials and methods used can be found in File S1.

Briefly, the expression stability of 22 widely used RG (Table S2 in File S1) was investigated across a total of 32 experimental settings with hepatocyte-like cell types, including freshly isolated primary human hepatocytes [5] at defined time points in cell culture (subgroup “primary hepatocytes”, PH), and HepG2 and Huh-7.5 cells treated with Chloroquine, Actinomycin D (ActD) [6], Trichostatin A [7] and DMSO - commonly used drugs with significantly differing effects in tissue culture - for different durations without passaging (subgroup “drug and density”, DD), or cultured for 14 days under a variety of conditions altering cell maturity status (subgroup “culture conditions”, CC) [8], [9] (all experimental settings listed in Table S1 in File S1). After RNA isolation and RT-qPCR, individual data sets of the samples, each containing Cycle Threshold (CT) values for all reference genes (primer details in Table S2 in File S1) and some exemplary genes of interest (target genes, TG; Table S3 in File S1) were further analysed *in silico*.

Similar to previous examinations of non-hepatic cell types [2], [3], the geNorm [10], Bestkeeper [11], and Normfinder [4] algorithms were used to evaluate and rank candidate RG.

## Results and Discussion

**Figure 1:**
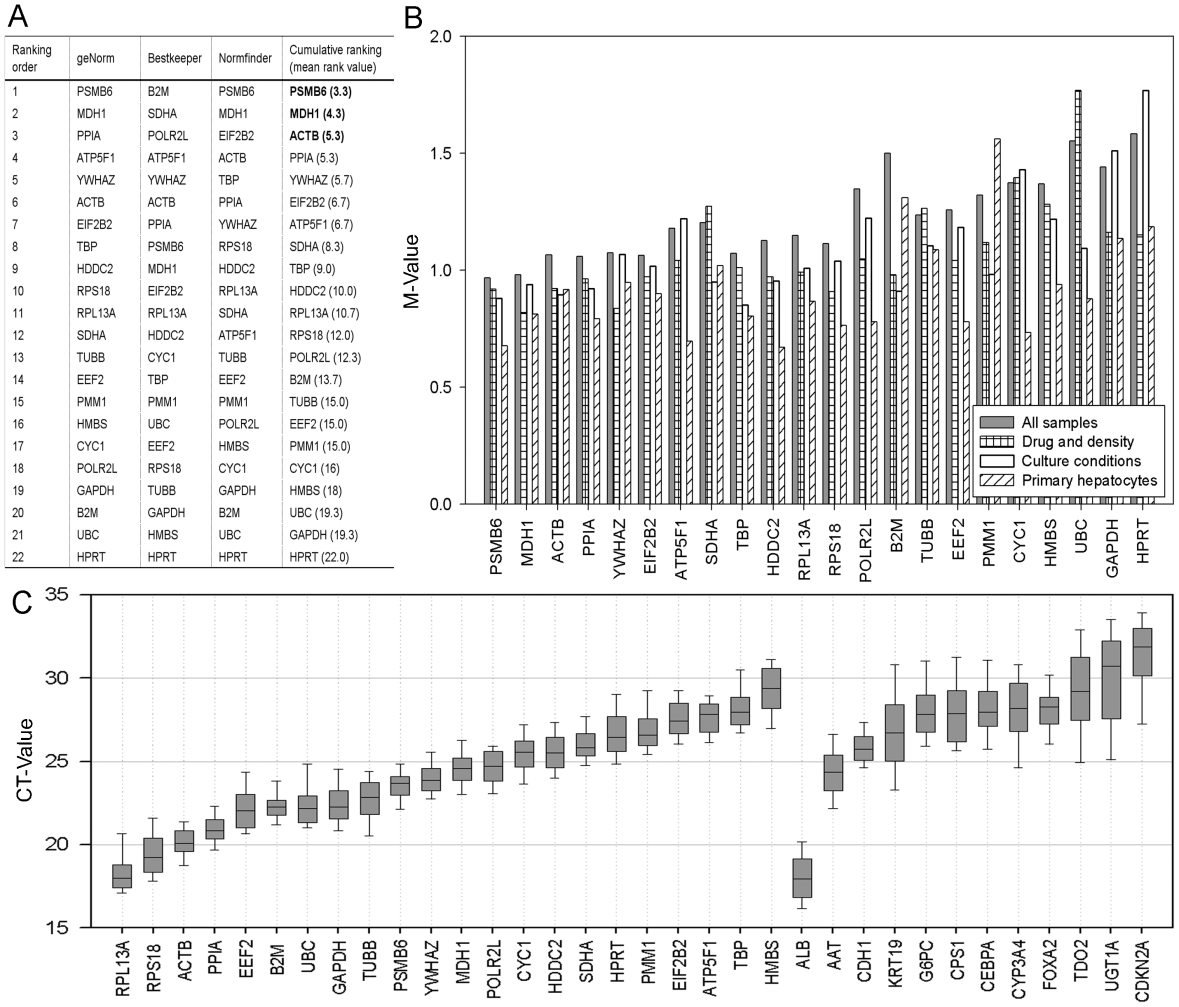
Evaluation and ranking of reference genes. (A) Overall ranking of 22 RG using geNorm, Bestkeeper and Normfinder algorithms and calculation of the cumulative ranking (column 4). (B) geNorm derived M-values as measures for the average pairwise expression stabilities within AS (column 1), and in the subgroups DD (2), CC (3) and PH (4). (C) Box-whisker plots of all CT-values of reference genes and target genes examined. Median (central horizontal line), the 25th and 75th quartile (boxes) and whiskers for the total CT-range are shown.

**Figure 2:**
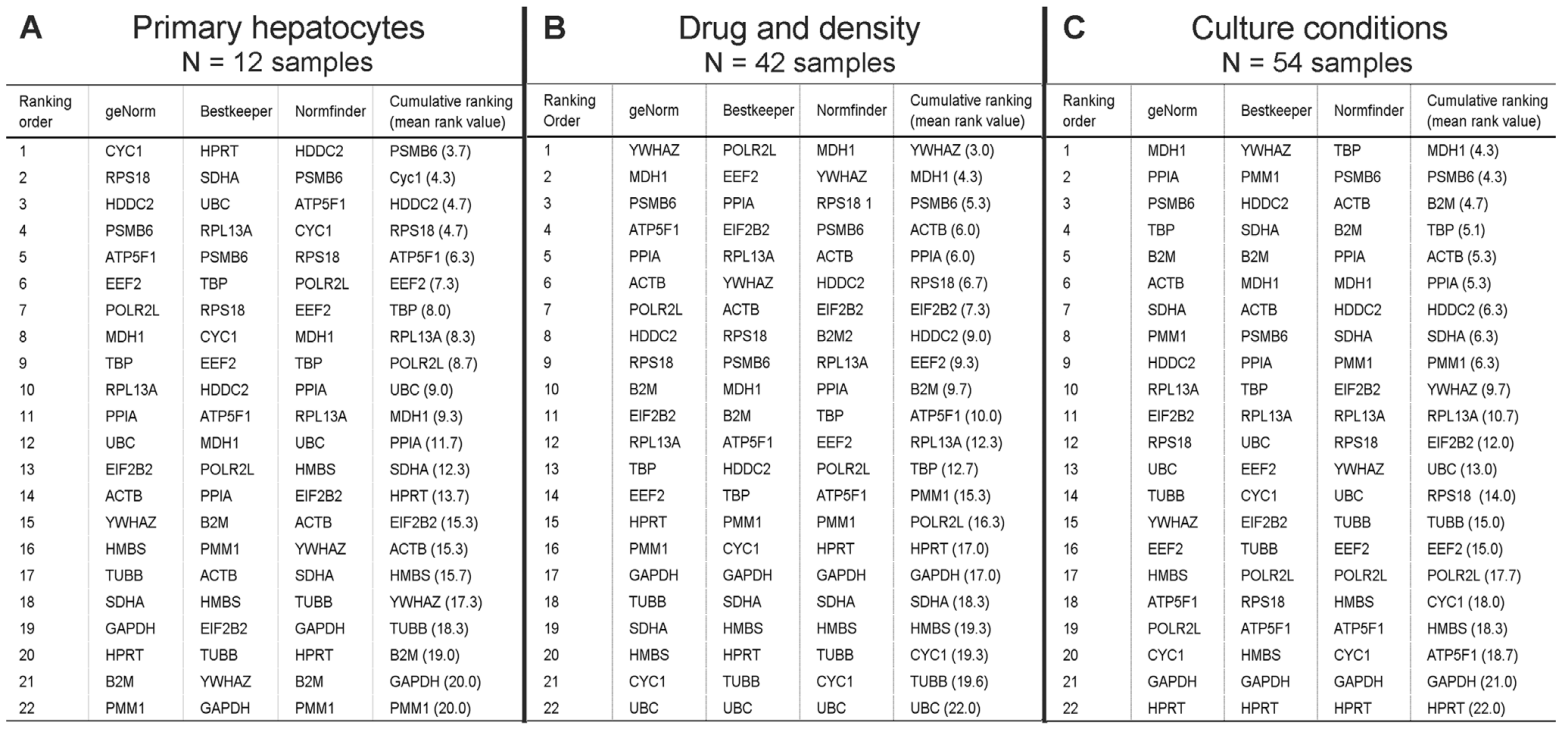
Reference gene rankings within the subgroups. (A) PH, primary hepatocytes, (B) DD, drug and density and (C) CC, culture conditions (also see Figure 1A, all samples).

The individual RG rankings (Fig. 1A (AS) and Fig. 2 (PH, DD, CC)), the analyses generated by the software algorithms (Fig. 1B, Fig. S1 in File S1), and the cumulative rankings should only serve as estimates: Individual softwares qualitatively differ to various extents - geNorm and Normfinder are more similar to each other than to Bestkeeper - hindering more exact combined ranking. Also, in the “all sample” group (AS), the three subgroups are not equally represented, in particular PH contributing only 12 data sets to a total of 96. However, from a general and overall point of view, a strikingly clear picture arises, even when taking into account that the PH rankings differ significantly from the cell line based analyses (DD and CC): *PSMB6* and *MDH1* could be identified as stable RG for all hepatocyte-like cells tested and some more genes (especially *ACTB*, *PPIA*, *HDDC2*) consistently rank well in the subgroups, offering the option to use multiple RG for normalisation. Noteworthy, albeit not included in the systematic analyses presented here, *PSMB1* and *MDH1* are also almost entirely unregulated in the human liver progenitor cell line HepaRG [12] under a variety of experimental conditions investigating metabolic activity or differentiation/maturation potential (data not shown).

Expression of the most commonly used *GAPDH* varies significantly in all subgroups and settings, similar to observations also in other cell types [13]–[15].

All RG identified as suitable are measured in a CT-range above 20 (Fig. 1C), thus are reasonably close to the ranges of TG CT-values - a prerequisite for accurate normalisation, mainly to cover the possibility of exhaustive processes in the course of thermal cycling.

**Figure 3:**
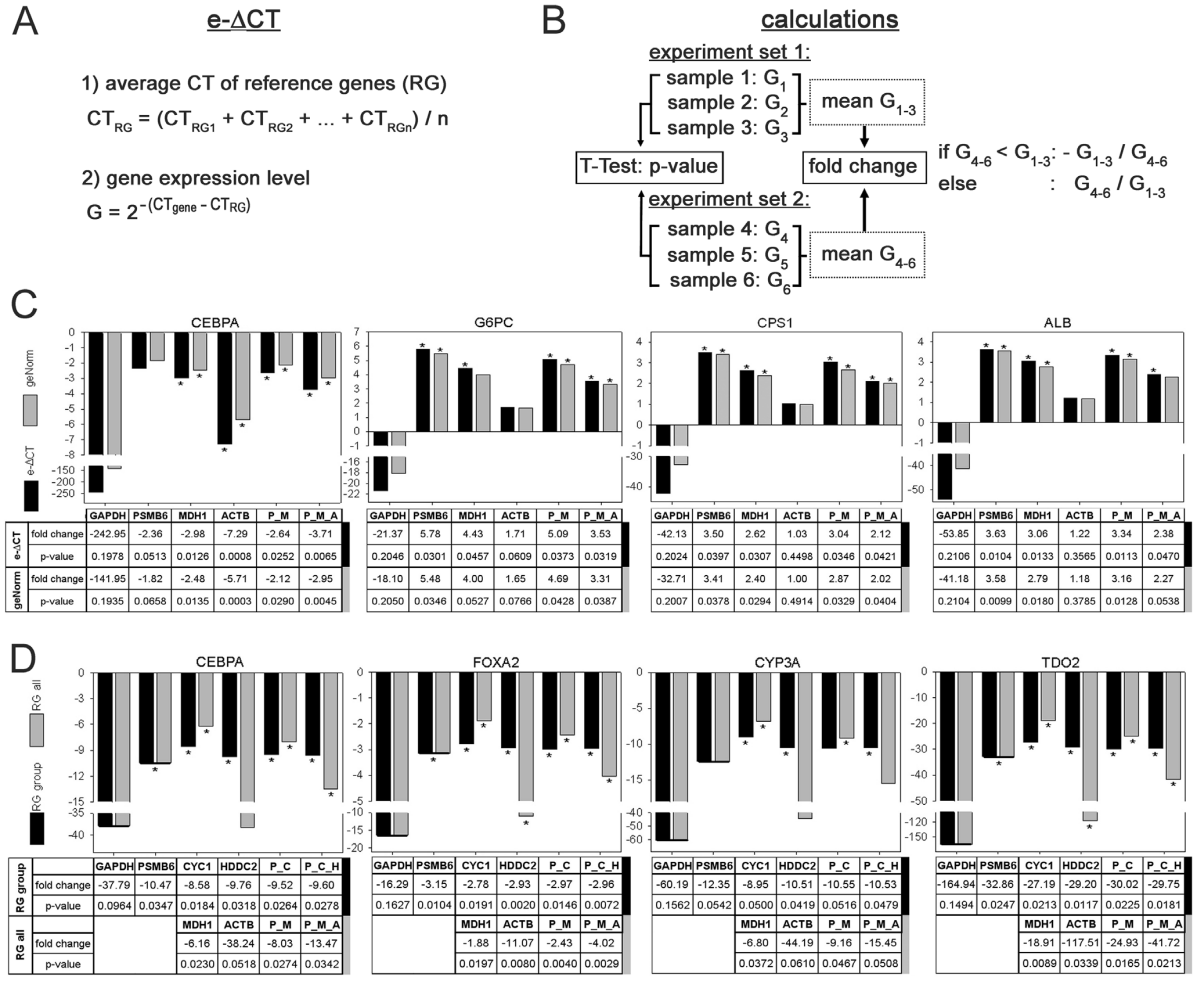
e-ΔCT-method and representative calculations of changes of target gene expression levels (expressed as fold change). (A) Calculation of e-ΔCT, an extension of the ΔCT-method by first calculating the mean CT-value of multiple RG included for each sample. (B) Depiction of data processing after geNorm-/e-ΔCT-calculations of sample-specific G-values to calculate fold changes and their significance (p-values) between two experimental settings, each consisting of three fully independent samples. (C) Example (Huh-7, without vs. with ActD) comparing fold changes in gene expression levels calculated by geNorm (grey) and e-ΔCT (black) using: GAPDH, the most common RG, the AS best ranking PSMB6, MDH1 and ACTB, and inclusion of two (PSMB6 and MDH1; P_M) or three (PSMB6, MDH1 and ACTB; P_M_A) RG. (D) Examples (primary hepatocytes, 0 vs. 24 hrs) comparing use of most stable RG of the subgroup (RG group, i.e. PH) vs. overall RG (RG all, i.e. AS) via e-ΔCT. *: p-values <0.05.

In many RT-qPCR applications relative changes of TG expression levels between experimental settings - and not absolute molecule numbers - are of interest. For such relative comparisons, the ΔCT method is most straightforward and often used [16], [17]. So far, the ΔCT-method does not allow for the inclusion of multiple RG. Thus we extended the ΔCT calculation (e-ΔCT) to permit the use of multiple RG (Fig. 3A): Since fold changes of expression levels are calculated as ratios of two experimental settings (Fig. 3B), the absolute CT-values in the two ΔCT-calculations of the ratio are cancelled. Only CT-differences between numerator and denominator in the fraction render essential.

Raw multiple RG CT-values obtained from one cDNA-sample can safely be regarded as being independent from each other with respect to choosing the method of calculating a mean CT-value. Thus, the arithmetic mean of the CTs of multiple RG is applied in e-ΔCT for variability-quenching and stabilisation purposes - with the advantages of using multiple RG discussed elsewhere [18] and also demonstrated by some of our examples: Even well-suited RG are regulated occasionally - thus not entirely stable - leading to misestimates of TG expression (Fig. 3D, Table S4 in File S1). For instance in PH, *PSMB6* and *CYC1* (Fig. 3D), both with similar M-values (Fig. 1B), lead to different results for the fold change - hence at least one has to be slightly regulated in primary hepatocytes within the first 24 hours of culturing. Further, regulation of *ACTB*, with a mediocre ranking in PH (Fig. 2) and obviously regulated, is sufficiently quenched by the other two RG (Fig. 3D, grey bars: ACTB vs. P_M_A). Thus, inclusion of multiple RG, which ideally serve independent cellular functions and therefore are least likely of being co-regulated in the same experimental systems, significantly buffers the effects of RG regulations.

In Fig. 3C (and Table S4 in File S1), e-ΔCT-derived expression level changes are compared with geNorm-derived values. geNorm, so far the only widely used method using multiple RG, estimates the absolute molecule numbers of a TG in a sample, but requires the availability of standard curve data that have to be established for each primer pair-template combination (with no fully defined method for generating standard curves being available [19], [20], but several methodical variants, each with its own accuracy-related shortcomings). ΔCT, not allowing determination of absolute mRNA molecule numbers, only requires the primer pairs chosen to be reasonably efficient - which can be verified quite easily [17]. However, although generated by significantly different underlying formulas, the outcomes of e-ΔCT and geNorm - using one, two or three RG - never differ by more than 30%, with similar significance measures (p-values) (Fig. 3C, Table S4 in File S1).

Whereas *PSMB1* and *MDH1*, ranking first in AS, appear suited also in all subgroups tested, sole use of *ACTB* would lead to misestimates, in particular in PH (Fig. 3D, Table S4 in File S1). However, we arbitrarily preferred *ACTB* to *PPIA* (same ranking in AS, Fig. 1A) as a third RG, for use together with *PSMB6* and *MDH1*: Inclusion of *ACTB* - its gene product being widely used as standard in other, non PCR-based assays - would allow to cross-compare qPCR findings with other assays where β-Actin is used for calibration.

Having analysed a reasonably wide spectrum of hepatic cell types and experimental conditions, this study aims to provisionally recommend a set of RG to be used as a routine and default choice for RT-qPCR analyses with human hepatocyte-like cells in general. However, caution is needed and RG stability should be verified [17] specifically for each experimental setting: Certainly many experiments exist that require a separate and specific search for appropriate RGs.

In conclusion, RG suited for RT-qPCR-analyses of hepatocyte-like cells *in vitro* could be identified. Two or three RG used with the straightforward e-ΔCT calculation can greatly improve reproducibility and robustness of relative gene expression data generated by RT-qPCR.

## Supporting Information

File S1 Supporting information. Containing Supplementary Materials and Methods, References, Figure S1, Tables S1, S2, S3 and S4.
(DOC)
