# Supplementary material for: An Extended ΔCT-Method Facilitating Normalisation with Multiple Reference Genes Suited for Quantitative RT-PCR Analyses of Human Hepatocyte-Like Cells
Source: PLoS One. 2014 Mar 21;9(3):e93031. doi: 10.1371/journal.pone.0093031 (PMC3962476; doi:10.1371/journal.pone.0093031)
Supplement: File S1 — Supporting information. Containing Supplementary Materials and Methods, References, Figure S1, Tables S1, S2, S3 and S4. (DOC) [file pone.0093031.s001.doc]

**Supplementary Materials and Methods**

**Cell culture**

*Huh-7 and HepG2:* Human hepatoma-derived cell lines Huh-7.5 and HepG2 were used for testing of culture conditions, drug toxicity- and density- studies. Huh-7.5 [1] cells were kindly provided by Thomas Pietschmann (Twincore, Hannover, Germany). HepG2 were received from DSMZ (Braunschweig, Germany). Both cell lines were maintained in basic Medium (BM), being DMEM with 2 mM L-Glutamin (PAA, Pasching, Austria) and 2% Penicillin/ Streptomycin (Biochrom,. Berlin, Germany) and supplemented with 10% FCS (PAN Biotech, Aidenbach, Germany).

*Primary human hepatocytes:* Hepatocyte isolation was performed using a modified 2-step collagenase perfusion technique as previously reported [2]. Briefly, the liver specimen obtained after partial hepatectomy was cannulated under sterile conditions and flushed once with 500 ml pre-warmed (37^o^C) washing buffer containing 2.5mM EGTA. This was followed by perfusion with 100 ml of a pre-warmed (37^o^C) digestion buffer containing 0.05% collagenase (Roche, Mannheim, Germany) allowing recirculation of the perfusate. Upon sufficient digestion, the tissue was mechanically disrupted and the emerging cell suspension poured through a gauze-lined funnel followed by centrifugation (50 g, 5 min., 4^o^C). The resulting cell pellet was washed twice using ice-cold PBS (50 g, 5 min., 4^o^C) and resuspended in William´s medium E (Biochrom) supplemented with 1 µM insulin, 1 µM dexamethasone/fortecortin (Sigma-Aldrich, Munich, Germany), 100 U/ml penicillin, 100 µg/ml streptomycin, 1 mM sodium pyruvate, 15 mM HEPES buffer (Biochrom), 4 mM L-Glutamine and 5% FCS. Cell number and viability were determined by the Trypan blue exclusion test.

For cell-size based separation, 10 ml of a 5 million cells/ml cell suspension were supplemented with 15 ml water and 5 ml Easycoll Separating solution (density 1.124 g/ml, Biochrom). The mixture was centrifuged in 4 °C for 20 minutes set to 1278 g with 0 deceleration. Supernatant was discarded and the pellet was resuspended in HCM (see below).

Hepatocytes were plated on Collagen I-coated (rat tail, Roche) 12 well plates with a cell density of 750,000 cells/well and cultured in HCM (Lonza; Basel, Switzerland; HBM with single quots of Ascorbic Acid, Hydrocortisone, Transferrin, Insulin, Gentamycin, BSA and rhEGF) supplemented with 10% Human Serum (AB-Serum, PAN Biotech). Medium was changed every second day. Cells were harvested immediately after isolation (0 h) and by trypsinization at 24, 72 and 168 hours after plating.

**Experimental settings, Huh-7.5 and HepG2**

*Culture conditions:* Huh-7.5 and HepG2 cells were cultured for two weeks under different conditions. 400,000 cells in BM per well were seeded in 12 well plates (Greiner, Frickenhausen, Germany) for adherence culture and in 24 well plates (ultra low attachment; Corning-Costar, Amsterdam, Netherlands) for suspension culture (promoting self assembly of cells and spheroid formation [3]) for 24hrs. Different conditions were set up afterwards as listed in Supplementary Table 1, using the ingredients as indicated: DMSO (Sigma-Aldrich, Munich, Germany), Oncostatin M (Peprotech, Hamburg, Germany), Dexamethasone (Sigma-Aldrich). Media were changed on the fourth and eighth day.

*Drug and Density:* 400.000 Huh-7.5 and HepG2 cells were plated in 12 well plates (Greiner) for 24hrs. Drug treatments and controls were set up afterwards as listed in Supplementary Table S1, using the ingredients as indicated and as published previously: Chloroquine (Carl Roth, Karlsruhe, Germany), Actinomycin D (AppliChem, Darmstadt, Germany) [4-7], Trichostatin A [8] and DMSO (Sigma-Aldrich).

**RNA Extraction and Reverse Transcription**

*RNA extraction*: Total RNA was extracted from cell pellets using the RNeasy Mini kit (Qiagen, Düsseldorf, Germany) according to the manufacturer’s instructions. Sample RNA concentrations were determined and only samples with an A260/A280 ratio between 1.8 and 2.0, indicating sufficient purity of the isolated RNA [9], were processed further.

*Reverse Transcription:* 1 µg RNA was treated with DNase and supplemented with Riboblock (Fermentas/VWR, Darmstadt, Germany) according to the manufacturer’s protocol. DNase-treated RNA was transcribed into cDNA using iScript (Bio-Rad, Munich, Germany) according to manufacturer´s instructions. Finally cDNA samples were diluted 1:10 with nuclease-free water (Ambion, Kassel, Germany) and stored at -20°C for qPCR use.

**Primers**

Twenty-two reference genes were chosen from the literature [10-17]. Primer sequences were taken from the literature (EIF2B2, EEF2, TBP [16], SDHA, HMBS, YWHAZ, RPL13A, UBC [11], CYC1 [14] and B2M [13]). If published primers resulted i) in low standard curve efficiency or ii) more than one unique RT-qPCR-amplicon from RNA-samples of our hepatic cell lines - as indicated by Gel-Electrophoresis and sequencing (see below), new primers were designed by NCBI/Primer-BLAST and Primer3 Input (version 0.4.0) softwares. Search parameters for NCBI/ Primer-Blast and Primer3 input were: Melting temperature (Tm) of 58–60°C, primer length of 20–24 nucleotides and a GC content of 50–60%.

PCR products for each primer pair were cloned into the pCR4-TOPO vector (Invitrogen) according to manufacturer’s instruction. Insert sequences were verified (SeqLab). TOPO constructs with PCR inserts were also used for generating standard curves (see below).

**qPCR**

qPCR was performed in a reaction volume of 25 µl, containing 1.9 µl cDNA samples, 400 nM of each primer (Sigma Aldrich), 12.5 µl Sybr Green I Reaction Mix (Bio-Rad) and nuclease-free water. All samples were measured in duplicates.

qPCR and accompanying melting curve analyses for validation purposes were performed using the LightCycler 480 (Roche) with settings as follows:

| 1.) Cycles | 1 | Mode | Heat activation |  |
| --- | --- | --- | --- | --- |
| Target (°C) | Acquisition Mode | Hold (hh:mm:ss) | Ram Rate (°C/s) | Acquisitions (per °C) |
| 95 | None | 00:05:00 | 4.4 |  |
| 2.) Cycles | 40 | Analysis | Quantification |  |
| Target (°C) | Acquisition Mode | Hold (hh:mm:ss) | Ram Rate (°C/s) | Acquisitions (per °C) |
| 95 | None | 00:00:10 | 4.4 |  |
| 55 | None | 00:00:10 | 2.2 |  |
| 72 | Single | 00:00:10 | 4.4 |  |
| 3.) Cycles | 1 | Analysis | Melting Curves |  |
| Target (°C) | Acquisition Mode | Hold (hh:mm:ss) | Ram Rate (°C/s) | Acquisitions (per °C) |
| 95 | None | 00:00:05 | 4.4 |  |
| 65 | None | 00:01:00 | 2.2 |  |
| 97 | Continuous |  | 0.11 | 5 |
| 4.) Cycles | 1 | Mode | Cooling |  |
| Target (°C) | Acquisition Mode | Hold (hh:mm:ss) | Ram Rate (°C/s) | Acquisitions (per °C) |
| 40 | None | 00:00:30 | 2.2 |  |

**Standard curves for determination of primer pair efficiency**

For the generation of standard curves all PCR-insert-containing TOPO constructs were pooled by mixing 10^10^ copies of each plasmid. From this pool, serial dilutions (containing10^8^-10^2^ copies per PCR-reaction) were used for qPCR with each primer pair. The PCR amplification efficiency of each primer pair was calculated using the equation:

Efficiency% = (10^[-1/slope]^ -1) x 100% [18].

Primer key data are listed in Supplementary Tables S2 and S3.

**Calculations and Statistical Analyses**

To identify the optimal reference gene among candidates, three different software tools – geNorm [11], Bestkeeper [19] and Normfinder [20] were used. Bestkeeper and geNorm determine the optimal reference gene by using a pair-wise correlation approach. Normfinder ranks reference genes by a model-based approach which also provides a calculation of intra- and intergroup variability. Cycle Thresholds (CT) of qPCR were determined by the LightCycler 480 Real-Time PCR System (Roche) using the Second Derivative Maximum. These raw data served as input for Bestkeeper. For geNorm and Normfinder CT-values were converted into total copy numbers of the PCR product by using standard curves data.

Bestkeeper (version 1) allows only 10 genes to be finally processed. Therefore, first standard deviations for all RG were calculated and the ten genes with the lowest standard deviation in each group were used further. Ranking of these ten genes was according to correlation coefficients calculated.

The geNorm (version 3.5) and Normfinder (Version 0.953) softwares used (pairwise) reference gene stabilities as the basis for their rankings.

For the cumulative rankings, combined from geNorm, Bestkeeper and Normfinder rankings, the arithmetic mean of all three positions of each reference gene was calculated for each group.

*Calculation of expression level changes for example target genes*

Gene expression regulation of example genes was calculated either by the e-ΔCT approach, a derivative of the ΔCT-method [21,22] (Fig. 3A) by simply using an averaged CT-value from mutiple reference genes instead of one reference gene-CT in ΔCT, or by geNorm [11]. Both approaches allow for the use of multiple RG.

First, expression levels G (see Fig. 3B) for each target gene and experimental data set based on the raw CT-values of one to three RG and of the TG were calculated. geNorm first calculates absolute expression levels (approximate molecule numbers) from CT-values using the standard curves established for each sample and gene primer pair, which are then divided by a normalisation factor derived basically from the geometric mean of the absolute expression levels of the RG included in sample qPCR-measurements. e-ΔCT (Fig. 3A) directly calculates a relative expression level for each TG per individual data set. For each experimental setting three fully independent data sets (individual experiments set up in separate weeks) were generated.

In a second step, the mean G-values from three independent gene expression levels G for each TG for each experimental setting, calculated either via e-ΔCT or via geNorm, served to obtain a (relative) value for expression level changes (“fold change”) - calculated as the ratio of two mean G-values.

Individual G-values (n=3 per experimental setting) were also used to perform a T-test to obtain p-values as a measure of statistical significance for the TG expression level changes between two experimental settings. p-values < 0.05 were considered significant.

**Supplementary References**

1. Blight KJ, McKeating JA, Rice CM. (2002) Highly permissive cell lines for subgenomic and genomic hepatitis C virus RNA replication. J Virol 76: 13001-13014.

2. Vondran FW, Katenz E, Schwartlander R, Morgul MH, Raschzok N, et al. (2008) Isolation of primary human hepatocytes after partial hepatectomy: Criteria for identification of the most promising liver specimen. Artif Organs 32: 205-213.

3. Tostoes RM, Leite SB, Serra M, Jensen J, Bjorquist P, et al. (2012) Human liver cell spheroids in extended perfusion bioreactor culture for repeated-dose drug testing. Hepatology 55: 1227-1236.

4. Deng R, Yang D, Yang J, Yan B. (2006) Oxysterol 22(R)-hydroxycholesterol induces the expression of the bile salt export pump through nuclear receptor farsenoid X receptor but not liver X receptor. J Pharmacol Exp Ther 317: 317-325.

5. Tapryal N, Mukhopadhyay C, Das D, Fox PL, Mukhopadhyay CK. (2009) Reactive oxygen species regulate ceruloplasmin by a novel mRNA decay mechanism involving its 3'-untranslated region: Implications in neurodegenerative diseases. J Biol Chem 284: 1873-1883.

6. Tan SH, Shui G, Zhou J, Li JJ, Bay BH, et al. (2012) Induction of autophagy by palmitic acid via protein kinase C-mediated signaling pathway independent of mTOR (mammalian target of rapamycin). J Biol Chem 287: 14364-14376.

7. Galmiche A, Ezzoukhry Z, Francois C, Louandre C, Sabbagh C, et al. (2010) BAD, a proapoptotic member of the BCL2 family, is a potential therapeutic target in hepatocellular carcinoma. Mol Cancer Res 8: 1116-1125.

8. Yamashita Y, Shimada M, Harimoto N, Rikimaru T, Shirabe K, et al. (2003) Histone deacetylase inhibitor trichostatin A induces cell-cycle arrest/apoptosis and hepatocyte differentiation in human hepatoma cells. Int J Cancer 103: 572-576.

9. Wilfinger WW, Mackey K, Chomczynski P. (1997) Effect of pH and ionic strength on the spectrophotometric assessment of nucleic acid purity. BioTechniques 22: 474-6, 478-81.

10. Radonic A, Thulke S, Mackay IM, Landt O, Siegert W, et al. (2004) Guideline to reference gene selection for quantitative real-time PCR. Biochem Biophys Res Commun 313: 856-862.

11. Vandesompele J, De Preter K, Pattyn F, Poppe B, Van Roy N, et al. (2002) Accurate normalization of real-time quantitative RT-PCR data by geometric averaging of multiple internal control genes. Genome Biol 3: RESEARCH0034.

12. Cicinnati VR, Shen Q, Sotiropoulos GC, Radtke A, Gerken G, et al. (2008) Validation of putative reference genes for gene expression studies in human hepatocellular carcinoma using real-time quantitative RT-PCR. BMC Cancer 8: 350.

13. Kadl A, Huber J, Gruber F, Bochkov VN, Binder BR, et al. (2002) Analysis of inflammatory gene induction by oxidized phospholipids in vivo by quantitative real-time RT-PCR in comparison with effects of LPS. Vascul Pharmacol 38: 219-227.

14. Heishi M, Kagaya S, Katsunuma T, Nakajima T, Yuki K, et al. (2002) High-density oligonucleotide array analysis of mRNA transcripts in peripheral blood cells of severe atopic dermatitis patients. Int Arch Allergy Immunol 129: 57-66.

15. Congiu M, Slavin JL, Desmond PV. (2011) Expression of common housekeeping genes is affected by disease in human hepatitis C virus-infected liver. Liver Int 31: 386-390.

16. Fedrigo O, Warner LR, Pfefferle AD, Babbitt CC, Cruz-Gordillo P, et al. (2010) A pipeline to determine RT-QPCR control genes for evolutionary studies: Application to primate gene expression across multiple tissues. PLoS One 5: e12545.

17. Synnergren J, Giesler TL, Adak S, Tandon R, Noaksson K, et al. (2007) Differentiating human embryonic stem cells express a unique housekeeping gene signature. Stem Cells 25: 473-480.

18. Bustin SA, Benes V, Garson JA, Hellemans J, Huggett J, et al. (2009) The MIQE guidelines: Minimum information for publication of quantitative real-time PCR experiments. Clin Chem 55: 611-622.

19. Pfaffl MW, Tichopad A, Prgomet C, Neuvians TP. (2004) Determination of stable housekeeping genes, differentially regulated target genes and sample integrity: BestKeeper--excel-based tool using pair-wise correlations. Biotechnol Lett 26: 509-515.

20. Andersen CL, Jensen JL, Orntoft TF. (2004) Normalization of real-time quantitative reverse transcription-PCR data: A model-based variance estimation approach to identify genes suited for normalization, applied to bladder and colon cancer data sets. Cancer Res 64: 5245-5250.

21. Livak KJ, Schmittgen TD. (2001) Analysis of relative gene expression data using real-time quantitative PCR and the 2(-delta delta C(T)) method. Methods 25: 402-408.

22. Schmittgen TD, Livak KJ. (2008) Analyzing real-time PCR data by the comparative C(T) method. Nat Protoc 3: 1101-1108.

**Supplementary Figure S1**

**
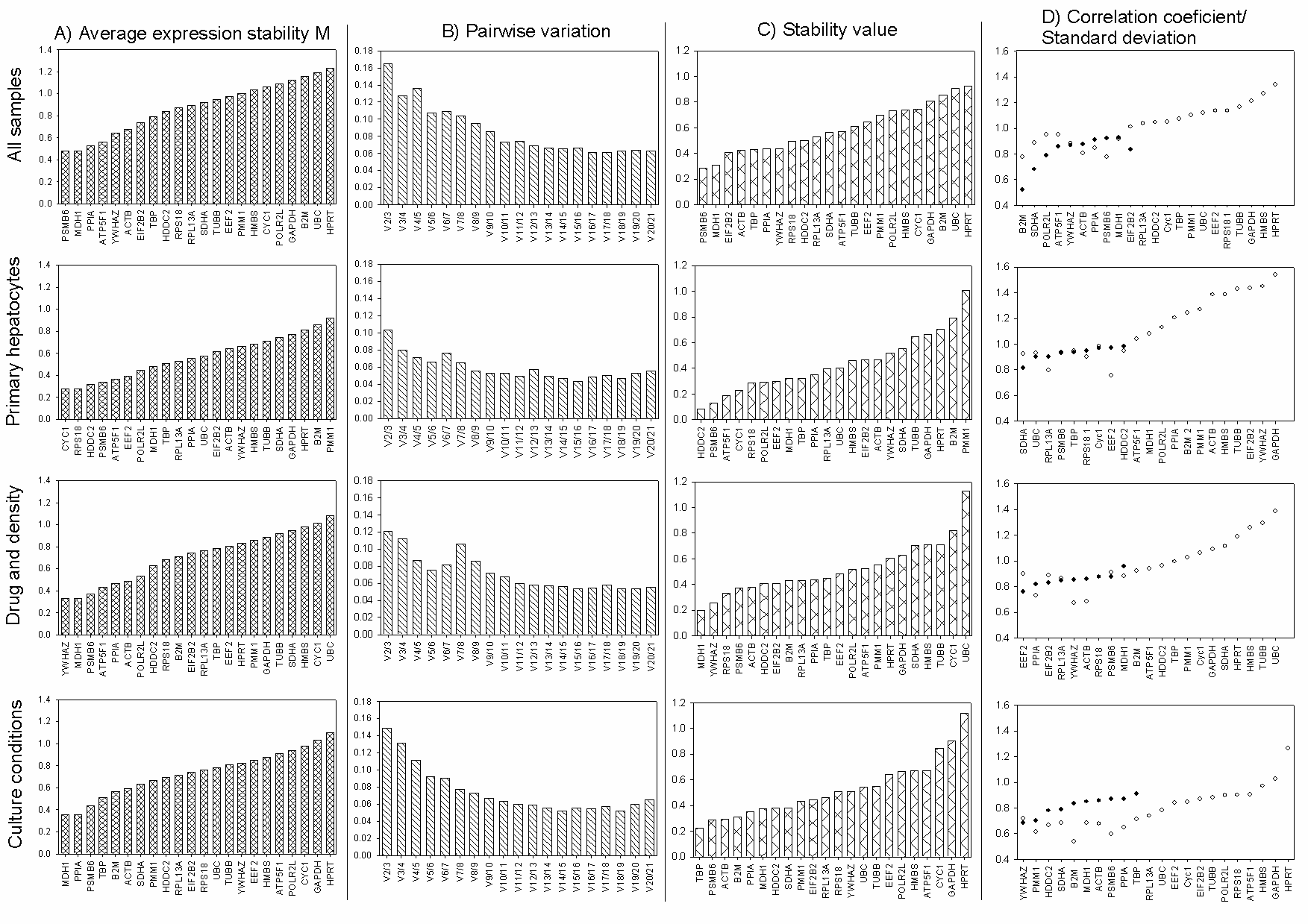
**

**Supplementary Fig. S1.** Key data of RG-analyses. (A) geNorm: Average expression stability: The RG ranking determined according to the average expression stability M in the AS group, and the PH, DD, and CC subgroups. A stepwise exclusion of the least stable RG was conducted to obtain the mean expression stability value M of the remaining RG until the two most stable RG were identified. (B) geNorm: pairwise Variation: Determination of the optimal number of control genes for normalisation based on pairwise variations. V-values for the significancy of the effect of adding another reference gene are plotted, with a cut-off value of 0.15. Thus, in the AS group three RG are recommended (V2/3 of 0.16), in all subgroups inclusion of two RG already appears sufficient (C) NormFinder: Gene expression stability values. Gene expression stability values of genes from the least stable (right) to most stable (left) (D) Bestkeeper: Calculation of correlation coefficient and standard deviation: Since Bestkeeper software allows only for the inclusion of ten candidate RG, first the standard deviations for the CT-values of each RG within each group were calculated. 12 RG with the highest standard deviation were ruled out and the remaining ten genes were used for the calculation with Bestkeeper. Open circles: Standard deviations; filled circles: Bestkeeper correlation coefficients.

**Supplementary Table S1**

Experimental settings (N = 32, 96 data sets)

| **#** | **Cell type** | **Medium** | **Serum** | **Supplements** | **Duration** | **Plate type** |
| --- | --- | --- | --- | --- | --- | --- |
| **Group: *Primary Hepatocytes* (PH) (N = 4, 12 data sets)** | | | | | | |
| **1** | Hepatocytes | HCM | HS |  | 0hrs | - |
| **2** | Hepatocytes | HCM | HS |  | 24hrs | Collagen |
| 3 | Hepatocytes | HCM | HS |  | 72hrs | Collagen |
| 4 | Hepatocytes | HCM | HS |  | 1week | Collagen |
| **Group: *Drug and Density* (DD) (N = 14, 42 data sets)** | | | | | | |
| 5 | HepG2 | BM | FCS |  | 24hrs | adherence |
| 6 | HepG2 | BM | FCS | 25µM Chloroquine | 24hrs | adherence |
| 7 | HepG2 | BM | FCS | 5µg/ml ActD | 24hrs | adherence |
| 8 | HepG2 | BM | FCS |  | 72hrs | adherence |
| 9 | HepG2 | BM | FCS | 250nM TSA | 72hrs | adherence |
| **10*** | HepG2 | BM | FCS |  | 2weeks | adherence |
| **11** | HepG2 | BM | FCS | 1% DMSO | 2weeks | adherence |
| **12** | Huh-7.5 | BM | FCS |  | 24hrs | adherence |
| 13 | Huh-7.5 | BM | FCS | 25µM Chloroquine | 24hrs | adherence |
| **14** | Huh-7.5 | BM | FCS | 5µg/ml ActD | 24hrs | adherence |
| 15 | Huh-7.5 | BM | FCS |  | 72hrs | adherence |
| 16 | Huh-7.5 | BM | FCS | 250nM TSA | 72hrs | adherence |
| **17** | Huh-7.5 | BM | FCS |  | 2weeks | adherence |
| **18** | Huh-7.5 | BM | FCS | 1% DMSO | 2weeks | adherence |
| **Group: *Culture conditions* (CC) (N = 18, 54 data sets)** | | | | | | |
| **19** | HepG2 | BM | FCS |  | 2weeks | adherence |
| **20** | HepG2 | BM | FCS | 1% DMSO | 2weeks | adherence |
| 21 | HepG2 | BM | FCS |  | 2weeks | suspension |
| 22 | HepG2 | BM | HS |  | 2weeks | suspension |
| 23 | HepG2 | BM | HS | 1% DMSO | 2weeks | suspension |
| 24 | HepG2 | HCM | HS | w/o rhEGF, 20ng/ml OSM, 4ng/ml Dex, | 2weeks | adherence |
| **25** | HepG2 | HCM | HS | w/o rhEGF, 20ng/ml OSM, 4ng/ml Dex, 1%DMSO | 2weeks | adherence |
| 26 | HepG2 | HCM | HS | w/o rhEGF, 20ng/ml OSM, 4ng/ml Dex | 2weeks | suspension |
| **27** | HepG2 | HCM | HS | w/o rhEGF, 20ng/ml OSM, 4ng/ml Dex, 1%DMSO | 2weeks | suspension |
| **28** | Huh-7.5 | BM | FCS |  | 2weeks | adherence |
| **29** | Huh-7.5 | BM | FCS | 1% DMSO | 2weeks | adherence |
| 30 | Huh-7.5 | BM | FCS |  | 2weeks | suspension |
| 31 | Huh-7.5 | BM | HS |  | 2weeks | suspension |
| 32 | Huh-7.5 | BM | HS | 1% DMSO | 2weeks | suspension |
| 33 | Huh-7.5 | HCM | HS | w/o rhEGF, 20ng/ml OSM, 4ng/ml Dex | 2weeks | adherence |
| 34 | Huh-7.5 | HCM | HS | w/o rhEGF, 20ng/ml OSM, 4ng/ml Dex, 1%DMSO | 2weeks | adherence |
| 35 | Huh-7.5 | HCM | HS | w/o rhEGF, 20ng/ml OSM, 4ng/ml Dex | 2weeks | suspension |
| 36 | Huh-7.5 | HCM | HS | w/o rhEGF, 20ng/ml OSM, 4ng/ml Dex, 1%DMSO | 2weeks | suspension |

**Supplementary Table S1.** All experimental settings. Each experiment was performed three times independently, resulting in 96 data sets from 32 experimental settings. Bold numbers: 4 settings included twice, for RG calculations in two groups. Bold and underlined: Settings used for representative calculations (Fig. 3C,D and Supplementary Table S4). Abbreviations: BM: basic medium: DMEM with 2 mM L-Glutamine and 2% Penicillin/Streptomycin; HCM: HBM (Lonza) with all single quots (Lonza: Ascorbic Acid, Hydrocortisone, Transferrin, Insulin, Gentamycin, BSA and rhEGF) added; FCS: Fetal Calf Serum, HS: Human Serum; ActD: Actinomycin D; TSA: Trichostatin A; DMSO: Dimethylsulfoxide; rhEGF: recombinant human Epidermal Growth Factor; OSM: Oncostatin M; Dex: Dexamethasone.

**Supplementary Table S2**

| **Gene** | **Full name** | **Acc.no.** | **Forward primer (5’ -> 3’)** | **Reverse primer (5’ -> 3’)** | **Slope** | **Y-intercept** | **R²** | **Efficiency (%)** |
| --- | --- | --- | --- | --- | --- | --- | --- | --- |
| **PSMB6** | Proteasome (prosome, macropain) subunit, beta type 6 | NM_002798 | CGGGAAGACCTGATGGCGGGA | TCCCGGAGCCTCCAATGGCAAA | -3.416 | 41.41 | 0.999 | 98.1 |
| **MDH1** | Malate dehydrogenase, transcript variant 1 | NM_001199111 | GTCACGACTGTGCAGCAGCGT | TGGGGTTCCAAACCAGATGTCCCTG | -3.589 | 43.87 | 0.999 | 95.0 |
| **ACTB** | Actin, beta | NM_001101 | ctcttccagccttccttcct | agcactgtgttggcgtacag | -3.615 | 43.44 | 0.999 | 94.5 |
| **PPIA** | Peptidylprolyl isomerase A (cyclophillin A) | NM_021130 | CCACCGCCGAGGAAAACCGTG | AAACAGCTCAAAGGAGACGCGGC | -3.219 | 40.16 | 0.993 | 102.2 |
| **YWHAZ** | Tyrosine tryptophan 5–monooxygenase activation protein, zeta | NM_001135699 | ACTTTTGGTACATTGTGGCTTCAA | CCGCCAGGACAAACCAGTAT | -3.568 | 42.02 | 0.999 | 95.3 |
| **EIF2B2** | Eukaryotic translation initiation factor 2B2 | NM_014239 | TCAAGATTATCCGGGAGGAG | ATGGAAGCTGAAATCCTCGT | -3.417 | 41.40 | 0.987 | 98.1 |
| **ATP5F1** | ATP synthase 5, Fo complex, subunit B1 | NM_001688 | TCGCGAGACTTGTGAGCGGCC | CCCGGGACAGCATGGTCAACG | -3.444 | 40.96 | 0.999 | 97.6 |
| **SDHA** | Succinate dehydrogenase complex, subunit A | NM_004168 | TGGGAACAAGAGGGCATCTG | CCACCACTGCATCAAATTCATG | -3.329 | 41.11 | 0.994 | 99.9 |
| **TBP** | TATA box binding protein (transcript variant 1) | NM_003194 | GCTGAGAAGAGTGTGCTGGA | GTAAGGTGGCAGGCTGTTGT | -3.464 | 41.62 | 0.999 | 97.2 |
| **HDDC2** | HD domain containing protein 2 | NM_016063 | GTCCAGAGGCCGGAGAGCGTT | TGGTGCTATGTCCCCAACGATGCA | -3.108 | 38.97 | 0.999 | 104.9 |
| **RPL13A** | Ribosomal protein L13a | NM_012423 | CCTGGAGGAGAAGAGGAAAGAGA | TTGAGGACCTCTGTGTATTTGTCAA | -3.516 | 42.13 | 0.999 | 96.2 |
| **RPS18** | Ribosomal protein S18 | NM_022551 | ACATCGATGGGCGGCGGAAA | CTCCCGCCCTCTTGGTGAGGT | 3.630 | 42.10 | 0.999 | 94.0 |
| **POLR2L** | Polymerase (RNA) II polypeptide L | NM_021128 | GGATGCTGCTGGCCCACGTG | CCCGGATGCCTCAGCCTCGT | -3.381 | 41.24 | 0.999 | 98.8 |
| **B2M** | Beta 2 microglobulin | NM_004048 | GATGAGTATGCCTGCCGTGTG | CAATCCAAATGCGGCATCT | -3.410 | 41.42 | 0.998 | 98.2 |
| **TUBB** | Tubulin, beta | NM_178014 | agtgccggaactcacccag | tcatcgacctccttcatgga | -3.347 | 41.04 | 0.999 | 99.5 |
| **EEF2** | Eukaryotic translation elongation factor 2 | NM_001961 | AGAAGCTGTGGGGTGACAG | GATCAGCTGGCAGAAGGTG | -3.156 | 40.72 | 0.993 | 103.7 |
| **PMM1** | Phosphomannomutase 1 | NM_002676 | ACCGGGGCCCACATCTGTGT | TGGCTGGGGACGGTTGTCCA | -3.396 | 40.94 | 0.999 | 98.5 |
| **CYC1** | Cytochrome c-1 | NM_001916 | GCTACACGGAGGATGAAGCTAA | ATAGTCGAACAGCTTCCCTGG | -3.406 | 40.87 | 0.999 | 98.3 |
| **HMBS** | Hydroxymethylbilane synthase | NM_000190 | GGCAATGCGGCTGCAA | GGGTACCCACGCGAATCAC | -3.207 | 41.15 | 0.999 | 101.1 |
| **UBC** | Ubiquitin C | NM_021009 | ATTTGGGTCGCGGTTCTTG | TGCCTTGACATTCTCGATGGT | -3.392 | 41.24 | 0.999 | 98.6 |
| **GAPDH** | Glyceraldehyde-3-phosphate dehydrogenase | NM_002046 | ctctggtaaagtggatattg | ctcccccctgcaaatgag | -3.540 | 43.65 | 0.998 | 95.8 |
| **HPRT1** | Hypoxanthine phosphoribosyl-transferase 1 | NM_000194 | Caggcagtataatccaaaga | atatcctacaacaaacttgt | -3.297 | 41.69 | 0.999 | 100.5 |

**Supplementary Table S2:** Reference genes: Full names, Genebank accession numbers, qPCR primer sequences, and standard curve data: Slope, y-intercept, coefficient of determination (R^2^) and efficiency.

**Supplementary Table S3**

| **Gene** | **Full name** | **Acc.no.** | **Forward primer (5’ -> 3’)** | **Reverse primer (5’ -> 3’)** | **Slope** | **Y-Intercept** | **R²** | **Efficiency (%)** |
| --- | --- | --- | --- | --- | --- | --- | --- | --- |
| **ALB** | Albumin | NM_000477.5 | TTGATTGCCTTTGCTCAGTA | GCCATTTCACCATAGGTTTC | -3.473 | 42.29 | 0.994 | 97.0 |
| **AAT** | Alpha-1 antitrypsin | NM_001127707.1 | AAGACAGATACATCCCACCA | AGGATTTCATCGTGAGTGTC | -4.264 | 52.25 | 0.999 | 85.8 |
| **CDH1** | Cadherin 1, type 1, E-cadherin | NM_004360.3 | AGGAATCCAAAGCCTCAGGT | CCCCGTGTGTTAGTTCTGCT | -3.197 | 41.50 | 0.998 | 102.8 |
| **KRT19** | Keratin 19 | NM_002276.4 | ctggttcaccagccggactg | atcagcgcctggatatgcgc | -3.012 | 41.63 | 0.985 | 107.4 |
| **G6PC** | Glucose-6-phosphatase, catalytic subunit | NM_000402.3 | atgcggttccagcctatct | tgcccccgaccgtctac | -3.125 | 41.92 | 0.992 | 104.5 |
| **CPS1** | Carbamoyl-phosphate synthase 1 | NM_001122633.2 | CCATCCCATAATTCCTGCTG | TCTCCAGTGGAAGCCATCTC | -3.474 | 43.17 | 0.999 | 97.0 |
| **CEBPA** | CCAAT/enhancer binding protein alpha | NM_004364.3 | agtggcggcagcggcgcgg | gctgcttggccttgtcg | -3.783 | 43.78 | 0.998 | 98.9 |
| **CYP3A** | Cytochrome P450, family 3, subfamily A | NM_017460.5 | accgtgacccaaagtactgg | gtttctgggtccacttccaa | -3.549 | 43.55 | 0.991 | 95.5 |
| **FOXA2** | Forkhead box A2 | NM_021784.4 | ccgttctccatcaacaacct | ggggtagtgcatcacctgtt | -3.116 | 42.74 | 0.994 | 104.5 |
| **TDO2** | Tryptophan 2,3-dioxygenase | NM_005651.3 | AAGAGGAACAGGTGGCTGAA | ATGCTCCCTGAAGTGCTCTG | -3.426 | 45.06 | 0.995 | 99.4 |
| **UGT1A** | UDP glucuronosyltransferase 1, polypeptide A | NM_001072.3 | GTGCCTTTATCACCCATGCT | GCTTTGCATTGTCCATCTGA | -3.421 | 44.11 | 0.991 | 98.0 |
| **CDKN2A** | Cyclin-dependent kinase inhibitor 2A | NM_000077.4 | gacctggctgaggagctg | ggatgtctgagggaccttcc | -3.276 | 45.93 | 0.956 | 101.0 |

**Supplementary Table S3:** Target genes: Full names, Genebank accession numbers, qPCR primer sequences, and standard curve data: Slope, y-intercept, coefficient of determination (R^2^) and efficiency.

**Supplementary Table S4**

**
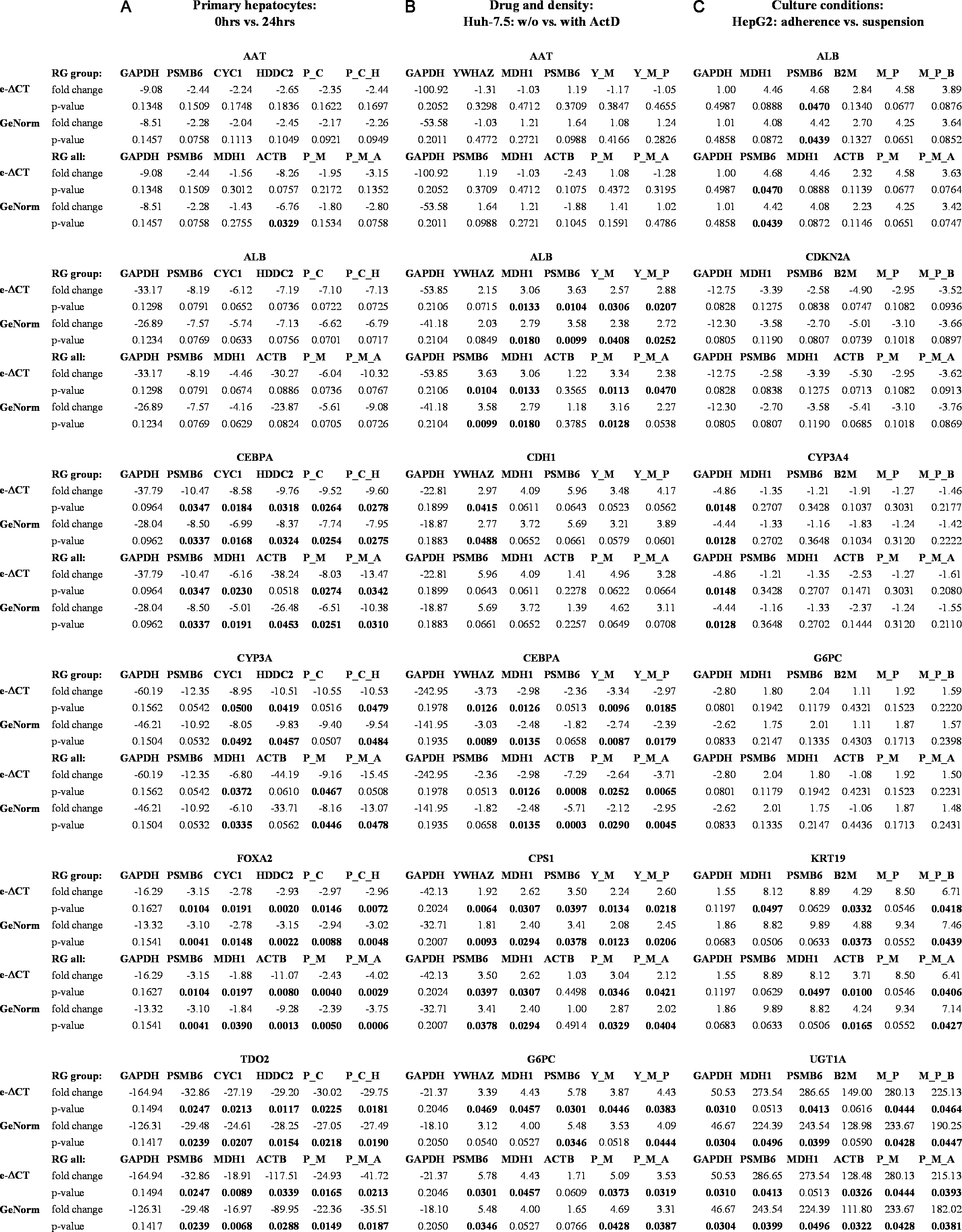
**

**Supplementary Table S4:** Representative calculations for fold changes of TG expression levels and significance measures (p-values)(see Fig. 3). Comparison of data derived via e-ΔCT and geNorm, normalised with GAPDH, the most commonly used RG, the three best RG genes of the corresponding subgroup (PH; DD, CC) and the overall best ranking RG (AS). p-values <0.05 in bold. (A) examples from subgroup “primary hepatocytes”, PH; (B) from subgroup “drug and density”, DD; (C) from subgroup “culture conditions”, CC.
